# Hematopoietic Stem Cell Regulation by Type I and II Interferons in the Pathogenesis of Acquired Aplastic Anemia

**DOI:** 10.3389/fimmu.2016.00330

**Published:** 2016-08-29

**Authors:** Julianne N. P. Smith, Vikramjit S. Kanwar, Katherine C. MacNamara

**Affiliations:** ^1^Department of Immunology and Microbial Disease, Albany Medical College, Albany, NY, USA; ^2^Department of Pediatrics, Division of Pediatric Hematology-Oncology, Albany Medical Center, Albany, NY, USA

**Keywords:** hematopoietic stem cells, interferon-gamma, interferon type I, aplastic anemia, bone marrow microenvironment, macrophages, T lymphocytes

## Abstract

Aplastic anemia (AA) occurs when the bone marrow fails to support production of all three lineages of blood cells, which are necessary for tissue oxygenation, infection control, and hemostasis. The etiology of acquired AA is elusive in the vast majority of cases but involves exhaustion of hematopoietic stem cells (HSC), which are usually present in the bone marrow in a dormant state, and are responsible for lifelong production of all cells within the hematopoietic system. This destruction is immune mediated and the role of interferons remains incompletely characterized. Interferon gamma (IFNγ) has been associated with AA and type I IFNs (alpha and beta) are well documented to cause bone marrow aplasia during viral infection. In models of infection and inflammation, IFNγ activates HSCs to differentiate and impairs their ability to self-renew, ultimately leading to HSC exhaustion. Recent evidence demonstrating that IFNγ also impacts the HSC microenvironment or niche, raises new questions regarding how IFNγ impairs HSC function in AA. Immune activation can also elicit type I interferons, which may exert effects both distinct from and overlapping with IFNγ on HSCs. IFNα/β increase HSC proliferation in models of sterile inflammation induced by polyinosinic:polycytidylic acid and lead to BM aplasia during viral infection. Moreover, patients being treated with IFNα exhibit cytopenias, in part due to BM suppression. Herein, we review the current understanding of how interferons contribute to the pathogenesis of acquired AA, and we explore additional potential mechanisms by which interferons directly and indirectly impair HSCs. A comprehensive understanding of how interferons impact hematopoiesis is necessary in order to identify novel therapeutic approaches for treating AA patients.

## Introduction

The concept of aplastic anemia (AA) was first introduced by Paul Ehrlich in 1888 and describes patients who fail to form blood cells from all three lineages, in association with decreased or absent bone marrow precursor cells. Although there are many known etiologies, the cause of AA is generally difficult to determine in an individual patient and in the vast majority of cases no causal etiology is found (1). The focus of the current review is on the role of interferons in the pathophysiology of this bone marrow failure (BMF) syndrome. The association of disease with expansion of autoreactive T lymphocytes (2, 3) and responsiveness of disease to immunosuppressive therapies, including antithymocyte globulin (ATG) and cyclosporine (4), demonstrate the immune-mediated nature of acquired AA. Although the precise cause of acquired AA is unknown, links to radiation, chemical exposure, and infection have been made. Gene polymorphisms that alter cytokine production or stability, particularly interferon gamma [IFNγ; Ref. (5)] provide additional evidence that dysregulated inflammatory responses are an essential driving force in the BMF seen in acquired AA. Mechanisms underlying the loss of hematopoietic stem cells (HSCs) during BMF include increased apoptosis and enhanced stem cell activity resulting in exhaustion. Here, we focus on the role(s) of interferons in the pathogenesis of BMF, and highlight new questions and avenues of research that may reveal therapies for targeted treatment of acquired BMF.

### Regulation of HSC Function: Intrinsic and Niche-Mediated Mechanisms

Quiescence preserves the self-renewal capacity and, therefore, the long-term function of HSCs. The regulators of this dormant state include intrinsic pathways as well as soluble and contact-dependent factors present in the niche microenvironment [reviewed in Ref. (6)]. Dysregulated HSC cycling may contribute to AA by enhancing differentiation over self-renewal or by sensitizing HSCs to apoptosis (7–10). Interferons have been implicated in both driving proliferation (11) and impairing proliferation of primitive hematopoietic stem and progenitor cells (HSPCs) (12), and sensitizing cells to apoptosis (13), thus supporting the notion that IFNs directly impair hematopoiesis by compromising stem cell function.

An altered microenvironment may also contribute to the pathogenesis of AA. Analysis of BM mesenchymal stromal cells (MSCs) derived from AA patients revealed reduced proliferative capacity and adherence, and a propensity to differentiate into adipocytes at the expense of osteoblasts (OBs) (14, 15). Considering the essential survival and dormancy-enforcing cues provided by niche cells, it will be important to investigate more fully the defects in stromal cells in acquired AA, and the impact of IFNs, either directly or indirectly, on such cells.

### Interferons in Acquired AA

The observation that patients with acquired AA exhibit increased levels of circulating IFNγ was made over 30 years ago (16). The presence of T cells containing intracellular IFNγ and positive for the prototypical Th1 transcription factor Tbet is an indicator of disease (17), and reduced frequencies of IFNγ positive T cells correlates with responsiveness to immunosuppressive therapy (4), suggesting that Th1 cells contribute to disease pathogenesis. Attempts to understand how IFNγ-mediated disease pathogenesis revealed that overexpression of IFNγ *in vitro* impairs long-term culture initiating cells LT-CIC (18), consistent with observations that neutralizing IFNγ in cultures derived from AA patients resulted in improved colony formation (16). Moreover, a polymorphism that results in enhanced stability of IFNγ transcripts is strongly associated with the risk of developing AA (5). However, the precise mechanisms whereby IFNγ drives BMF *in vivo* are still unclear and may involve multiple overlapping pathways and multiple cell types.

Type I IFNs (IFNα/β) are key regulators of innate and adaptive immunity. Although not directly implicated in AA pathogenesis, type I IFNs mediate host responses to most infections and contribute to autoimmunity in systemic lupus erythematosus [recently reviewed in Ref. (19)] and potentially in diabetes mellitus, Sjogren’s syndrome, autoimmune myositis, and rheumatoid arthritis (20, 21). Pegylated IFNα 2a (PEG-IFNα2a) is the standard of care in hepatitis C virus (HCV) patients, but is also a treatment option for melanoma (22), hairy cell leukemia (23), and multiple sclerosis (24–26). Type I IFN therapy is not well tolerated by all patients, however, and hematologic side effects are closely monitored. HCV patients receiving both PEG-IFNα2a and the nucleoside analog ribavirin are prone to hemolytic anemia due to ribavirin processing in erythrocytes as well as PEG-IFNα2a-mediated BM suppression (27, 28). Rarely, type I IFN therapies have also been linked to persistent BM suppression and the development of AA (24, 29, 30). BM suppression appears not to require exogenous or supraphysiologic levels of IFNα/β, as anemia and BM failure have also been associated with physiologic type I IFN responses to chronic viral infection (31). Of particular relevance to AA, the impact of type I IFNs on hematopoiesis is often not immediately suppressive, but requires secondary stress, such as exposure to subsequent IFNγ during the pathogenesis of lymphocytic choriomeningitis virus (LCMV) infection (12). Herein, we will discuss the potential for direct and niche-mediated type I IFN stimulation to impair HSCs and contribute to acquired AA.

### Bone Marrow Failure Induced by Infection

Bone marrow suppression has been observed subsequent to a number of viral infections, including parvovirus (32, 33), human immunodeficiency virus [HIV; Ref. (34)], viral hepatitis (35), Epstein–Barr virus (36), and influenza (37), among others. The ability of viral infections to suppress the BM may be due to both the ability of viruses to actively infect cells of the hematopoietic system and the host response to the virus, likely involving production of interferons and other pro-inflammatory factors. BM suppression and severe cytopenias are also common after exposure to tick bites, and are associated with the rickettsial pathogens *Ehrlichia chaffeensis* and *Anaplasma phagocytophilum* (38). Though transient, cytopenias are often severe, and infection requires antibiotic treatment (39). Human monocytic ehrlichiosis has been associated with bone marrow hypoplasia (40) and hemophagocytic lymphohistiocytosis [HLH; (41)], and murine models implicate interferon responses in mediating bone marrow suppression in rickettsial infections (42–44).

### Models to Study Human AA

Bone marrow failure pathogenesis was first modeled in mice using exposure to toxins, instigated by the association of benzene exposure with human disease (45). Observation that AA is a result of immune-mediated pathology prompted the development of donor lymphocyte infusion models relying on the adoptive transfer of lymph node or spleen-derived lymphocytes from histocompatibility mismatched strains of mice (46). This model recapitulates many observations in human AA patients as protection can be achieved with immunosuppressive treatment and abrogation of IFNγ (47, 48). A technical hurdle of the infusion-based model is that the use of F1 recipients precludes analysis of genetically targeted mice. Thus, it has been difficult to evaluate direct and indirect roles of specific cytokines on hematopoietic versus stromal cells. However, it has allowed a deeper understanding of T cell intrinsic mechanisms necessary for initiation of disease, including Notch signaling (49) and transcriptional regulators of Tbet (50). To model human patients carrying a mutation that renders a higher risk for developing AA, a mutation was introduced to the 3′ untranslated region of the *Ifng* gene, stabilizing IFNγ transcripts (51). Termed “ARE-delete,” this mouse model reproduces many features of human disease and is not associated with autoreactive T cells, suggesting that elevated IFNγ, independent of activated T cells, can drive disease by impairing progenitor cell function (51). In addition, a number of insights into bone marrow suppression have come from studying bacterial and viral pathogens. In ehrlichiosis, HSC loss requires IFNγ sensing by macrophages, demonstrating that interferon signaling reduces the HSC supportive capacity of niche cells during infection-induced BM suppression (42). In LCMV, phenotypic HSCs are reduced early in the course of infection, independent of IFNγ and likely through the actions of type I IFNs (12, 31). Together, the observations made in murine infection models and in a subset of patients undergoing PEG-IFNα2a treatment provide additional evidence that interferons impair HSCs, likely via multiple direct and niche-mediated mechanisms.

## Mechanisms of IFNγ-Mediated AA

### HSC-Intrinsic Impact of IFNγ

The negative impact of IFNγ on hematopoiesis is well documented [reviewed in Ref. (52)], but what is the evidence that there is a direct impact of IFNγ on the most primitive HSCs? HSC loss can occur via impaired self-renewal, increased differentiation, or induction of cell death, which may be results of both direct and/or indirect effects of IFNγ. While some studies suggest that IFNγ has an antiproliferative effect on HSCs (12, 53), evidence also suggests that IFNγ signaling promotes proliferation, and subsequent exhaustion of HSCs (11, 54). During infection with *Mycobacterium avium* or LCMV, IFNγ increased HSC proliferation and led to a reduction in transplantable myeloid potential (11, 55). Moreover, HSCs from a microenvironment deficient in IFNγ have more robust long-term potential, whereas excessive IFNγ signaling reduces transplantable HSPC activity (54, 56), further suggesting that tonic IFNγ signaling limits HSC function, perhaps through inducing proliferation. The discordant results with respect to whether IFNγ induces or suppresses proliferation is further confounded by the complex interaction with other cytokines, as IFNγ can augment the expansion of myelogenous leukemia cells when it signals in concert with IL-3, but can suppress proliferation in cells lacking IL-3 stimulation (57). In addition, TNFα stimulation is necessary for maximal IFNγ-induced suppression and proliferation of leukemia cell cultures (57), further emphasizing the potential for IFNγ to elicit distinct and even opposing effects dependent on the local cytokine milieu.

**Figure 1:**
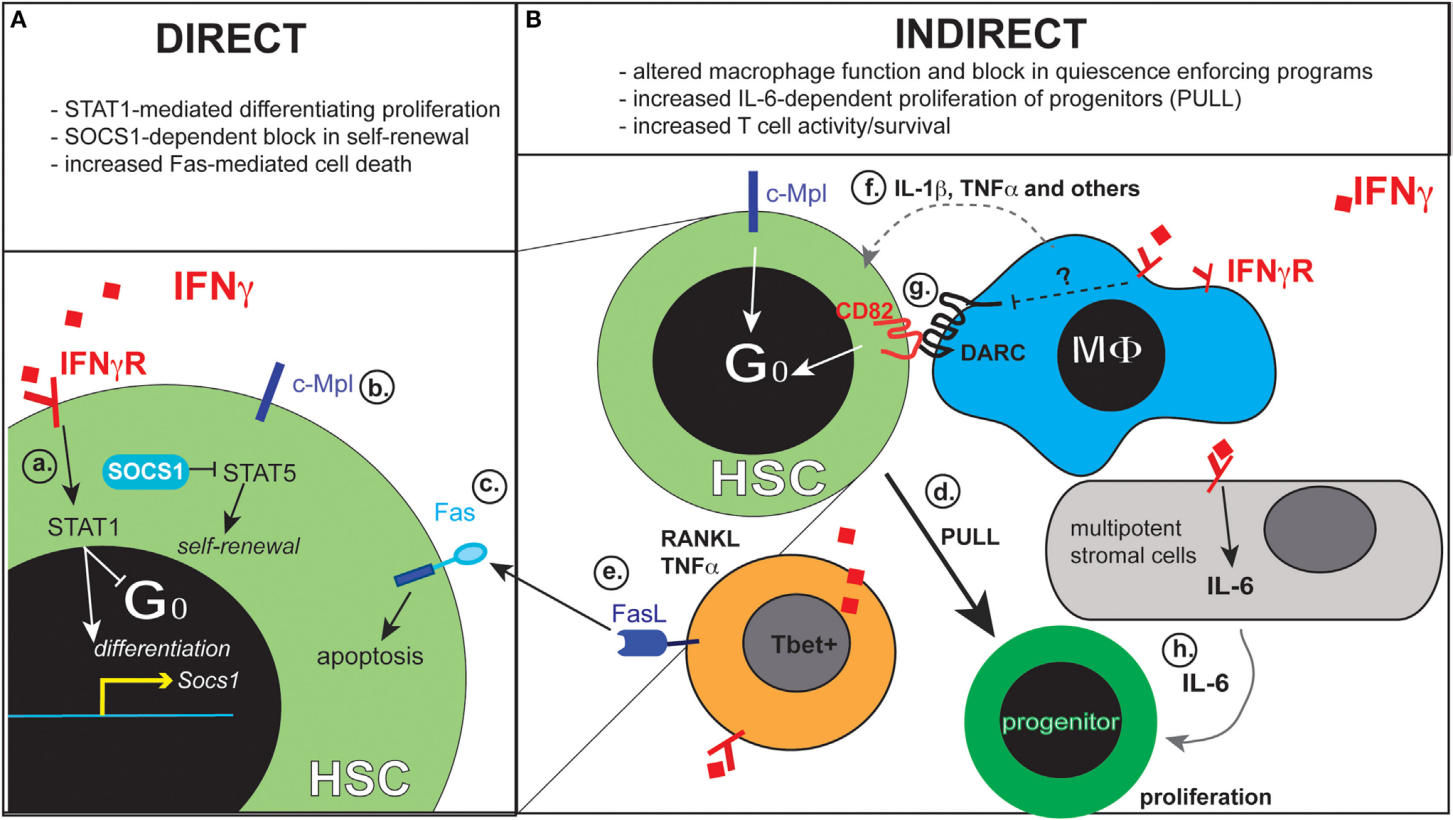
**The actions of IFNγ directly on HSCs and on cells of the microenvironment can result in HSC impairment in acquired aplastic anemia**. This figure summarizes key direct **(A)** and indirect **(B)** impacts of IFNγ on HSCs. The inset on the left depicts HSC-intrinsic effects of IFNγ, including STAT1-mediated hematopoietic differentiation programs (a), restriction of thrombopoietin – c-Mpl signaling by SOCS1 (b), and promotion of Fas expression (c). The inset on the right depicts cell types in the bone marrow microenvironment that are capable of regulating HSCs in an IFNγ-dependent manner include Tbet^+^ T lymphocytes, macrophages (MΦs), mesenchymal stromal cells, and hematopoietic progenitors. Known molecular mechanisms by which these cell types engage in IFNγ-dependent HSC regulation include: increased demand for progenitor cell differentiation to replenish downstream hematopoietic compartments (d), expression of death receptor ligands FasL and TNFα by T lymphocytes (e), propagation of MΦ-derived inflammatory signals (f) and potential impairment to MΦ-dependent regulation of HSC quiescence (g), and the production of further myelopoiesis-promoting factors by BM stromal cells (h).

Stem cell proliferation can result in the generation of more stem cells (self-renewal) or more committed progenitors (differentiation) and IFNγ has also been implicated in impeding self-renewing divisions (Figure 1a) (12, 58). Notably, IFNγ was shown to directly reduce HSC self-renewal during recovery from viral infection where robust type I IFNs had ablated the HSC pool (12), suggesting that type I IFNs may potentiate the suppressive impact of IFNγ on hematopoiesis during viral infection. These data highlight the importance of the cellular and cytokine context in the impact of single cytokines. Whereas IFNγ may not impede self-renewal in the steady state, prior exposure to type I IFNs may sensitize HSCs to the effects of IFNγ; at the same time, the induction of cellular stress by type I IFN-induced HSC cycling could enhance the potential for IFNγ to provoke HSC apoptosis during immune-mediated BM failure (59, 60).

Hematopoietic stem cells require a variety of inputs from growth factors, chemokines, G protein-coupled receptors, and cytokines to maintain their dormant status, location, and capacity to self-renew. An intriguing role for IFNγ in limiting responsiveness to the growth factor thrombopoietin (TPO) via the increase in suppressor of cytokine signaling (SOCS1) (12) (Figure 1b) illustrates yet another direct mechanism whereby IFNγ can impede HSC function. Support for the role of TPO in HSC function comes from the promising clinical data using a TPO receptor (c-Mpl) agonist, Eltrombopag (61). When given in combination with immunosuppressive drugs, it can provide tri-lineage recovery in patients refractory to traditional therapies (62). Though precise mechanisms of Eltrombopag function have not yet been elucidated, one possibility is that the drug works by overcoming a direct impact of IFNγ on suppressing TPO signaling in HSCs.

Interferon gamma is elicited by many microbial infections and plays a critical role in host defense by sensitizing cells to undergo apoptosis, thus impeding pathogen growth (63, 64). IFNγ can induce apoptosis by increasing the expression of Fas on cells subsequently targeted by Fas ligand-expressing cells, such as T lymphocytes (60). Evidence that HSCs express Fas in response to IFNγ (Figure 1c) suggests Fas-mediated destruction of HSCs contributes to their loss in AA (60). It is important, however, to consider the question of HSC sensitivity to IFNγ. Indeed, whereas some cell types respond very rapidly to IFNγ *in vitro*, such as macrophages, HSCs exhibit a much more subtle response, as measured by activation of STAT1 (42). This may indicate that the ability of HSCs to respond to IFNγ *in vivo* may be concentration dependent, and it suggests that HSCs are likely not first responders to IFNγ during an initial exposure. Under prolonged conditions of chronic exposure, however, HSCs may become direct targets. Thus, there are temporal considerations when evaluating the direct impact of IFNγ on HSCs under different inflammatory conditions.

### Impact of IFNγ on Progenitors

The idea that HSC activation can be achieved directly or as a result of demand implies that HSC loss may result from increased progenitor cell activity or loss. Several lines of evidence support a direct role for IFNγ in impacting murine progenitor cells in the context of infection (43, 65). IFNγ promotes the emergence of a phenotypically unique, hybrid progenitor population that expresses the IL-7 receptor and has both myeloid and lymphoid potential *in vitro*, but has primarily myeloid potential *in vivo*. Similarly, an intrinsic requirement for IFNγ was found to occur during bacterial infection, directing the production and terminal differentiation of myeloid cells (43). In a model of sterile inflammation, via adoptive transfer of activated effector T lymphocytes, IFNγ acted directly on progenitors, but not HSCs (66). The ability of IFNγ to act on downstream progenitors to drive proliferation, however, may indirectly call HSCs from a dormant state, which may explain observations suggesting that IFNγ acts directly on HSCs (Figure 1d).

### Indirect or Niche-Mediated Effects of IFNγ on HSC Function

#### T Lymphocytes

Although T cells are the cellular source of IFNγ that drives AA pathology, T cells also sense and respond to IFNγ. The effects of IFNγ on T cells include promotion of Th1 CD4^+^ T cell differentiation, enhancement of CD8^+^ T cell response, and subversion of IFNγ-mediated apoptosis via the downregulation of IFNγ receptor (67–69). IFNγ also primes activated T cells to secrete more abundant TNFα and RANKL (70, 71), inflammatory cytokines capable of inducing hematopoietic cell death and further inflammation. Additionally, T cells derived from AA patients show elevated Fas ligand expression (72) (Figure 1e), which is IFNγ dependent in murine lymphocyte infusion models of AA (60). Thus, it is likely that IFNγ acts to expand and preserve pathologic T cells in the BM during AA.

A population of T regulatory cells (Tregs) reside in the BM at homeostasis [reviewed in Ref. (73)] and establishes HSC-protective niches during transplantation and reconstitution (74). The direct HSC supportive capacity of Tregs in AA has not yet been evaluated, but Tregs derived from AA patients are reduced in number and inhibitory capacity, and show enhanced production of cytokines, including IFNγ (75, 76), suggesting that they may further contribute to immunopathology in the BM microenvironment. In Tregs, SOCS1 signaling controls IFNγ production (77) and defects in SOCS1 activity are thought to underlie autoimmunity and susceptibility to endotoxemia [reviewed in Ref. (78)]. Therefore, *ex vivo* expansion and treatment of autologous Tregs with small molecule SOCS1 mimetics may prove a promising therapeutic strategy for AA patients who do not respond well to conventional immunosuppression (76, 79).

#### Osteoclasts

Osteoclasts (OCLs) are bone-resorbing myeloid cells that are both directly and indirectly sensitive to IFNγ. Osteoclastogenesis requires the sensing of M-CSF and RANKL by myeloid precursors (80). Direct IFNγ sensing by myeloid precursors attenuates RANK signaling (81), but systemic IFNγ responses are associated with enhanced bone resorption due to the OCL-promoting impact of TNFα and RANKL (70, 71). Since IFNγ and TNFα levels are elevated in AA patients (82, 83), accelerated osteoclastic differentiation of myeloid precursors may occur early in AA pathogenesis. Indeed, low bone mineral density and osteoporosis are prevalent in individuals with the inherited BM failure condition Shwachman–Diamond syndrome (84) and have been observed in Fanconi anemia patients following BM transplantation (85). Whether inflammatory bone loss contributes to hematologic impairment in AA is currently unknown. OCLs and bone resorption have been found to reduce HSPC support in murine models, however, and are associated with HSPC mobilization (86, 87). The actions of bone-forming OBs and bone-resorbing OCLs are regulated primarily by the endocrine system (88). Since the responses of BM T cells to circulating hormones stimulates bone formation and short-term HSC expansion through Wnt signaling (89), T cell-based therapies warrant further investigation for their potential not only to reduce immunopathology, as mentioned above, but also to regenerate HSPCs and BM microenvironmental function in AA.

#### Macrophages

The BM microenvironment contains a heterogeneous population of tissue-resident macrophages (MΦs) that sense and respond to IFNγ [reviewed in Ref. (90)]. IFNγ stimulates MΦ cytokine production (Figure 1f) and antigen presentation (91), therefore, it stands to reason that MΦs may contribute to IFNγ-driven AA pathogenesis. We have previously established that MΦs in general, and IFNγ-stimulated MΦs in particular, reduce the pool of HSCs in a model of human monocytic ehrlichiosis, which causes transient BM suppression (42). Intriguingly, one of the few hematopoietic cell types found to be maintained in AA BM is the CD169^+^ MΦ (92). Tissue-resident MΦ populations, including BM-resident MΦs, are thought to be embryonically derived and maintained via self-renewal, rather than derived from HSC differentiation (93) [and recently reviewed in Ref. (94)]. This would support the idea that the maintenance of MΦs may not require an intact HSPC pool, thus explaining their persistence in the BM of patients with AA.

As antigen-presenting cells, MΦs are relatively weak (95), thus, it is unlikely that MΦs drive AA pathogenesis by activating T cells directly. Mice deficient in myeloid lineage cells are resistant to severe AA induction (96), however, suggesting that MΦs are indispensable in AA pathogenesis. While further investigation is necessary to determine if MΦ number and function correlates with AA severity, it can be envisioned that MΦs play a pathologic role in AA via several mechanisms. Since HSPCs and resident MΦs interact within the BM microenvironment (97–99), it is possible that IFNγ stimulates pathologic HSPC engulfment by MΦs in AA. In fact, IFNγ is associated with hemophagocytosis-induced anemia (100), and MΦs have been implicated in the pathogenesis of human hemophagocytic disorders, such as juvenile idiopathic rheumatoid arthritis and lymphohistiocytosis (101, 102), as well as platelet clearance in immune-mediated thrombocytopenia (103). Alternatively, MΦs may contribute to HSPC loss in AA by regulating, either directly or indirectly, HSPC proliferation or differentiation. Quiescent HSCs are called to proliferate and differentiate in response to demand for mature progeny, such as myeloid cells or platelets (104, 105), but must reenter quiescence in order to avoid replication stress and ensure lifelong maintenance. MΦs have been implicated in maintaining long-term HSC quiescence, or dormancy, through the production of PGE_2_ and the maintenance of the quiescence-promoting tetraspanin CD82 on the surface of HSCs via Duffy antigen receptor (DARC) on MΦs (98, 99) (Figure 1g); however, functional changes to this cell–cell interaction upon inflammation have only just begun to be investigated. In conditions of inflammation and infection, MΦs may suppress dormancy as a way to enlist HSCs in demand-adapted hematopoiesis. In murine ehrlichiosis, IFNγ is required for BM-resident MΦ maintenance, and is also essential for the infection-dependent loss of HSCs. Upon MΦ depletion, HSCs proliferate, under both steady-state (98) and infectious conditions (42). McCabe et al. found that these HSCs subsequently reenter quiescence, resulting in HSC pool expansion. Thus, under infection states, and perhaps in AA, IFNγ-stimulated MΦs drive HSC loss. This may occur via inhibition of HSC proliferation and demand-adapted hematopoiesis or alternatively, via increased differentiating proliferation, at the expense of self-renewal, culminating in HSC exhaustion.

Hematopoietic stem cells are motile within the BM of infected mice (106), suggesting that HSC engagement with the niche may be important for demand-adapted hematopoiesis. Since MΦs support the expression of HSPC retention factors by endosteal cells (97), MΦs may render HSCs more susceptible to T cell-mediated killing, and less capable of migration to microenvironments that support cell cycle entry and differentiation. At homeostasis, a population of endosteal MΦs, termed osteomacs, is reported to mediate osteoblastic NF-κB signaling, maintenance of bone-lining OBs, and hematopoietic progenitor cell retention in the BM (97, 107). Whether MΦs stimulated with IFNγ or other inflammatory cytokines, as in AA, drive osteoblastic dysfunction (see below), remains an open question. Since BM MΦs persist in AA patients, in spite of reductions in nearly all other BMC populations (92), and since MΦs potently respond to IFNγ, studies focused on the impact of MΦs in AA pathogenesis are warranted.

#### Mesenchymal Stromal Cells

Mesenchymal stromal cells respond to inflammatory signals, including IFNγ, to regulate the differentiation of HSCs and the mobilization of their progeny (66, 108). Cytotoxic CD8^+^ T cell-derived IFNγ was recently found to stimulate IL-6 production by BM MSCs, thus identifying a niche-mediated mechanism by which IFNγ stimulates myeloid transcriptional programs in hematopoietic progenitors (Figure 1h) (66). Consistent with these observations, BM stromal cells derived from AA patients and from a murine model of AA show elevated *Il6* expression (15, 109). Since there is a higher prevalence among AA patients for an Il6 gene polymorphism conferring IL-6 hypersecretion (83), it is currently unclear whether elevated IL-6 in AA is IFNγ-dependent. IL-17 is increased in the BM plasma of AA patients and more potently stimulates IL-6 secretion by MΦs derived from AA BM than from healthy controls (110), suggesting that inflammation in AA primes the responses of MSCs and other cell types to amplify local cytokine production. Since MSCs exist in close proximity to HSCs, and can greatly influence HSC fate, the impact of IFNγ on MSCs in AA is a key unanswered question in the field.

Adult BM MSCs are rare but exhibit heterogeneity with respect to their developmental origin, localization in the BM, and contribution to bone formation and HSPC regulation (111–114). This heterogeneity, coupled with the need for genetic reporter strains to identify and delineate MSC populations, has hindered investigation of BM MSCs in disease models, including in lymphocyte infusion-based AA models where IFNγ is known to be pathogenic. MSC dysfunction may contribute to BM failure, as MSCs possess immunoregulatory potential [reviewed in Ref. (115–117)] and are critical HSC-support cells. With regard to HSC niche function, peri-arteriolar MSCs enforce quiescence and are required for long-term HSC function (118). When the niche is activated, such as through hormonal stimulation, MSCs increase in number and mediate expansion of the HSC pool (112). Although adherent BM stromal cells, enriched in MSCs, show normal surface marker expression in AA patients, these cells fail to expand readily in culture, undergo greater apoptosis, and are impaired in osteogenic but enhanced in adipogenic differentiation, relative to normal controls (14, 15, 119). Unlike osteolineage cells, which support HSPCs and B lymphopoiesis (120, 121), BM adipocytes are detrimental to HSCs (122). MSC differentiation into either adipogenic or osteogenic progenitors is controlled by cell intrinsic and extrinsic mechanisms (123, 124). Systemic inflammation, as induced by high-fat diet, was recently linked to PPARγ activation in MSCs and resultant adipogenesis, concomitant with a reduction in HSPC support by the microenvironment (125). Severe AA, therefore, could erode BM microenvironmental function and HSC niches by a similar mechanism.

Elevated IFNγ may impact MSCs in AA via a number of distinct or overlapping mechanisms. T cell-mediated MSC killing, IFNγ-induced MSC dysfunction, or bystander effects mediated by neighboring BM cell types all potentially contribute to AA pathogenesis. Although MΦs are dispensable for the maintenance of BM MSCs at homeostasis, they regulate MSC function by promoting MSC expression of the niche-retention factors *Cxcl12, Angpt1, Kitl*, and *Vcam1* (126). Moreover, MΦs support the presence of mature OBs along the endosteum at homeostasis (97), potentially by stimulating NF-κB-mediated osteoblastic differentiation of MSCs or immature OBs (107). These data indicate that in an otherwise unperturbed system, MΦs promote the survival and/or osteolineage differentiation of bone-lining OBs. Thus, the potential for MΦs to dysregulate MSCs resulting in HSC niche destruction in AA and other disease states warrants investigation.

## Mechanisms of IFNα/β-Mediated HSPC Impairment

### HSC-Intrinsic Impact of Type I IFNs

Early observations made in LCMV-infected mice (31), and in IFNα-treated HSPC cultures (127), led to the conclusion that type I IFNs suppress progenitor cell proliferation and differentiation. Indeed, IFNα induces HSPC expression of cell cycle inhibitors *in vitro* (13). *In vivo*, however, the impact of IFNα/β differs. Acute administration of the double-stranded RNA mimetic polyinosinic:polycytidylic acid (polyI:C) causes rapid, IFNα receptor (IFNαR)-dependent HSC cycling (13, 128), and has been the model of choice for studying type I IFN-mediated HSPC activation (see Table 1 for a summary of relevant findings). The impact of polyI:C-induced sterile inflammation varies depending upon the duration of stimulation and the precise HSC subset analyzed, but acute stimulation is sufficient to decrease HSC expression of cyclin-dependent kinase inhibitors and quiescence-enforcing transcriptional programs, including FoxO3a, Notch, and TGFβ (13).

**Table 1:** **Impact of acute and chronic polyI:C-induced inflammation on HSCs and HSPCs**.

	HSCs	Hematopoietic progenitors
Acute	Reduced in frequency but not changed in number (13) Cell cycle entry (13, 59) Increased redox stress, accumulation of DNA double-strand breaks, and engagement of Fanconi anemia DNA repair pathway (59) Increased translation of megakaryocyte- lineage proteins (104) Enhanced death *in vitro* (59)	Increased myeloid (13) and CD41^hi^ stem-like megakaryocyte progenitors (104) No change in Lineage^−^ c-Kit^+^ cell cycling, DNA damage, or colony formation (59)
Chronic	Reduced in frequency, trend toward reduction in number (13) Loss of function in response to chemotherapeutic injury, transplantation, and *in vitro* expansion (13, 59, 128) Transiently reduced cyclin-dependent kinase inhibitor and quiescence-enforcing gene expression (13) Activation of PI3K/mTOR signaling (129) and increased m-Myc protein levels (130) Caspase 3 activation (13) Myeloid bias in transplantation (59)	Transiently increased Lineage^−^ c-Kit^+^ cell pool (13) Exhaustion of stem-like megakaryocyte progenitor cell function (104)

*Note that polyI:C was obtained from InvivoGen (59, 128, 130), GE Life Sciences (131, 132), and Invitrogen (104)*.

The function of type I IFNs in the context of physiologic induction, such as infection, may provide insight into pathogenic role(s) of type I IFNs in AA. Somewhat paradoxically given the BM suppressive impact of IFNα in viral infection, the type I IFN response to opportunistic *Pneumocystis* lung infection is protective in *Rag^−/−^* mice (133). Since these mice lack all B and T lymphocytes, immunity depends entirely upon myeloid cells, which undergo greater apoptosis in the absence of type I IFNs (134). In *Pneumocystis*-infected *Rag* competent mice, IL-10 and IL-27 production by B lymphocytes is protective, and correlates with enhanced myelopoiesis (135). Thus, in the absence of lymphocytes, and the cytokines they produce, type I IFNs provide a survival signal for myeloid cells. These findings further support the notion that the complex cytokine milieu greatly impacts the outcome of IFN signaling on HSC function.

At homeostasis, HSC quiescence protects against replication stress and genomic instability. Long-term label retaining studies demonstrate that ~1% of phenotypic HSCs cycle per day (136) and that a subset of multipotent progenitors is maintained in a similarly dormant state (137). PolyI:C increases HSC cycling six- to sevenfold for up to 3 days, leading to the accumulation of reactive oxygen species and DNA damage in remaining HSCs (59). DNA damage itself induces type I IFN-mediated stem cell senescence (138), in addition to the activation of cellular checkpoints and tumor suppressor genes [recently reviewed in Ref. (139)]. Additionally, type I IFNs transcriptionally regulate p53 (140), through the interferon-stimulated signaling complex ISGF3 (141, 142). Therefore, type I IFNs have the capacity to induce both proliferation as well as DNA damage-induced p53 signaling, thus priming HSCs to undergo apoptosis upon cellular stress, such as *in vitro* culture (13). These findings, therefore, implicate type I IFNs in the induction of replication and oxidative stress in HSCs.

*In vivo*, repeated IFNα/β stimulation or uncontrolled type I IFN signaling is detrimental to HSCs exposed to chemotherapeutic injury or transplantation (13, 128, 143), likely by promoting cell cycle entry and heightened sensitivity to cellular stress. While this may be detrimental in some cases, complete molecular remission has been observed in several cases of chronic myelogenous leukemia (CML) where IFNα pre-treatment was followed by imatinib mesylate (144), suggesting that IFNα may induce CML stem cell exit from dormancy and subsequent sensitization to growth factor withdrawal. The sensitizing effect of type I IFNs on stem cells persists, as HSCs transplanted from mice 2 weeks after polyI:C stimulation remained functionally impaired in their repopulating capacity (13). Therefore, it is reasonable to expect that increases in endogenous IFNα/β during viral infections or chronic administration of type I IFNs may have long-term impacts on HSC response to subsequent inflammatory stimuli. Such a mechanism is consistent with HSC impairment and BM suppression during LCMV infection, which elicits initial IFNα/β followed by subsequent IFNγ (12, 31, 145). Further studies are necessary, however, to determine if apoptosis is the predominant mechanism by which HSCs are depleted upon type I IFN sensitization, or if IFNα/β also sensitizes HSCs to senescence, or to non-apoptotic cell death.

In addition to HSC proliferation and apoptotic sensitization, type I IFNs influence HSPC differentiation. IFNα/β increases the synthesis of proteins required for rapid hematopoietic progenitor cell differentiation in response to inflammation and demand. This occurs via a post-transcriptional mechanism, the targets of which include the c-Myc transcription factor (130), and megakaryocyte lineage proteins (104). Expression of the megakaryocyte lineage gene von Willebrand factor and the alphaIIb integrin protein CD41 have previously been attributed to the most primitive HSCs (146, 147), but Haas et al. identified the IFNαR-dependent emergence of highly proliferative, CD41^hi^ megakaryocyte-restricted progenitor cells within the phenotypic HSC pool upon stimulation of mice with polyI:C, TNFα, or lipopolysaccharide (104). Since CD41 was not interrogated in previous studies, it is unclear to what extent megakaryocyte-primed progenitor cells contributed to the observed effects of type I IFNs on HSPC proliferation, apoptotic sensitization, and multilineage repopulation (13, 59, 128).

Hematopoietic stem cell metabolism is exquisitely regulated to protect against metabolic stress and to regulate the nature of cell division upon entry into the cell cycle (148), such as occurs upon type I IFN stimulation. One mechanism by which HSC metabolism is regulated is through autophagy and the Foxo family of transcription factors (149). FOXO3A, in particular, has been implicated in the activation of autophagy gene expression programs in HSCs that are essential for HSC survival upon cytokine withdrawal or calorie restriction-induced stress (150). Additionally, Warr et al. found that HSCs derived from aged mice had greater autophagic flux and were more reliant on autophagy for colony formation *in vitro*. Sterile type I IFN stimulation reduces FOXO3A expression and signaling activity in HSPC subsets (13, 104). Moreover, infection-induced type I IFNs are linked to reduced autophagic flux in the liver (151). The impact of type I IFNs on HSC autophagy has not yet been assessed, but autophagic suppression was recently identified in CD34^+^ BM cells from AA patients, and persisted even upon amelioration of AA symptoms (152). Therefore autophagy inhibition could represent an additional mechanism by which interferon signaling impairs HSC stress responses and exacerbates pathology in AA.

### Indirect or Niche-Mediated Effects of IFNα/β on HSC Function

#### T Lymphocytes

In AA pathogenesis, oligoclonally expanded CD8^+^ T cells infiltrate the BM and produce damaging, pro-inflammatory cytokines, including IFNγ (153). Type I IFNs regulate T cell production of IFNγ in a highly context-dependent manner, whereby type I IFNs are associated with enhanced IFNγ during extracellular bacterial infection (154), but with reduced IFNγ in response to intracellular pathogens (155–157). Type I IFNs may also contribute to the activation and expansion of pathologic T cells in AA as IFNα/β increases the survival of antigen-specific CD8^+^ T cell clones, as well as the generation and cytolytic activity of memory T cells (158, 159). Indeed, the blood and BM of AA patients show increased effector memory T cells (160), which may be derived from a newly identified class of progenitors termed memory stem T cells (161, 162). Since type I IFNs drive cell cycle entry and differentiation of other HSPC subsets (13, 104), they may also impact the development of CD8^+^ memory T cells from memory stem T cells and contribute to the etiology of infection-induced and iatrogenic BM failure through the modulation of T cell populations.

#### Macrophages

During infection, MΦs are stimulated concurrently with IFNs and TNFα and amplify inflammation through the production of additional IFNα/β and TNFα (163). Like IFNγ, TNFα is highly pathogenic in AA (164–166) and may engage in cross-talk with type I IFN signaling. TNFα levels correlate with the extent of cytopenia (165), and TNFα neutralization improves the colony-forming activity of AA patient BM (164). In addition to elevated circulating TNFα, TNF receptor 1 and 2 (TNFR1/TNFR2) expression is increased on hematopoietic progenitors derived from AA patients relative to healthy controls (165). TNFR2 ligation initiates inflammatory signaling, whereas TNFR1 drives the assembly of cytoplasmic cell death signaling complexes [reviewed in Ref. (167)]. A number of mechanisms, including caspase activity and ubiquitination of TNF receptor interacting protein kinase 1 (RIPK1), promote immunologically silent apoptosis when TNFR2 is activated [reviewed in Ref. (168)]. If caspase activity is limited, however, RIPK1–RIPK3 interactions mediate RIPK3-dependent phosphorylation of the pseudokinase MLKL. Phosphorylated MLKL then translocates to cellular membranes where its pore-forming action leads to cell lysis and the release of intracellular contents in a process known as necroptosis.

MΦs in *Salmonella typhimurium*-infected mice were the first cell type found to undergo IFNαR- and RIP1-dependent necroptosis *in vivo* (169). Subsequent studies have found an absolute requirement for IFNα/β priming in the death of MΦs by necroptosis (163, 170). Thus, the combinatorial impact of TNFα and type I IFNs has the potential to drive MΦs necroptosis in autoimmune diseases, such as severe AA. BM-resident MΦs are abundant HSPC–niche cells [recently reviewed in Ref. (90)], therefore, even a low rate of MΦ necroptosis has the potential to exacerbate immunopathology through release of damage-associated molecular patterns from the lytic cells, and/or impairment of the mononuclear phagocyte system responsible for clearing dead and dying cells, including apoptotic HSPCs. Further research is also needed to discern whether myeloid progenitors, and HSPCs themselves, have the capacity to undergo necroptosis in response to IFNα/β.

#### Stromal Cells

Although IFNα/β have not been directly implicated in AA pathogenesis, TNFα stimulates autocrine type I IFN expression in MΦs and in endothelial cells (171, 172), and could, therefore, establish local IFNα/β gradients in the inflamed BM microenvironment. Type I IFN sensing by BM stromal cells is not required for IFNα-induced HSPC proliferation in response to polyI:C, but *Ifnar1^−/−^* HSPCs are induced to proliferate in 95% WT: 5% *Ifnar1^−/−^* mixed BM chimeras (128), suggesting that IFNα/β-stimulated hematopoietic cells release additional factors that act on *Ifnar1^−/−^* HSPCs. HSPC-activating cues may derive from the HSPC pool itself, as hematopoietic progenitors produce a wide repertoire of inflammatory cytokines upon toll-like receptor stimulation (173), or may originate from stromal niche cells within the BM microenvironment.

Arteriolar blood vessels and megakaryocytes comprise HSC niches in the BM (174–176), although they are reported to be spatially and functionally distinct from one another. Sterile, IFNα/β-driven inflammation relocates HSPCs away from quiescence-enforcing arteriolar niches (118), though it is unclear whether this is cause or consequence of changes in HSC cycling. IFNα/β can also stimulate endothelial chemokine expression, including that of CCL5 or RANTES (177), which can impact platelet production by megakaryocytes (178). The role of megakaryocytes in HSC regulation is dynamic, as homeostatic expression of CXCL4 and TGFβ1 promotes quiescence, while concomitant increases in FGF-1 and decreases in TGFβ1 facilitate regeneration (174, 175). To our knowledge, megakaryocyte dysfunction has not been investigated in the pathogenesis of BM failure but aberrant TGFβ1 signaling is linked to pathologic extracellular matrix deposition and derangement of hematopoiesis in myelofibrosis (179). Additionally, TGFβ slows recovery from chemotherapy-induced myelosuppression by blocking HSC proliferation (180). Since type I IFNs both impair HSCs and activate a program of enhanced megakaryocyte lineage differentiation (104), it is intriguing to consider the impact this may have on HSPC–niche cell interactions during recovery from severe IFN-driven inflammation.

## Conclusion

In severe AA, autoreactive T cells initiate immunopathology, leading to HSC depletion, and total hematopoietic collapse. IFNγ is well-known to correlate with AA disease severity in mice and humans, but the mechanisms by which IFNγ impairs HSCs remain somewhat elusive. The potential for IFNγ to both directly exhaust and deplete HSCs, as well as to indirectly reduce HSC function through microenvironmental niche cells, particularly macrophages, and MSCs (Figure 1), adds complexity to the study of AA pathogenesis but also reveals new potential therapeutic targets. Since type I IFNs have been linked to BM aplasia and sensitize HSCs to cellular stress (Table 1), it can be envisioned that initial IFNα/β exposure, as occurs in response to viral infection, may render HSCs more vulnerable to subsequent IFNγ-mediated impairment. Current understanding of how inflammatory signals impact the HSC niche is limited; thus, we discussed several potential mechanisms by which interferons may contribute indirectly to HSC loss during severe AA. Parallels emerge when considering the impact of IFNγ and IFNα/β on HSCs, including the capacity of both cytokines to (1) drive HSC proliferation, seemingly at the expense of long-term function; (2) propagate inflammatory signaling within macrophages, a critical HSC niche cell type; and (3) potentiate cell death through the regulation of death receptor signaling, suggesting that these factors may be synergistically detrimental in inflammatory disease states. The development of additional AA mouse models, in which the independent and concerted impact of interferon signaling on specific cell types can be interrogated, would be of great utility in parsing out the mechanisms that drive AA pathogenesis.

## Author Contributions

All authors listed, have made substantial, direct and intellectual contribution to the work, and approved it for publication.

## Conflict of Interest Statement

The authors declare that the research was conducted in the absence of any commercial or financial relationships that could be construed as a potential conflict of interest. The reviewer MB and handling Editor declared their shared affiliation, and the handling Editor states that the process nevertheless met the standards of a fair and objective review.
